# Imaging Findings of a Rare Intrahepatic Splenosis, Mimicking Hepatic Tumor

**DOI:** 10.3390/diagnostics15141789

**Published:** 2025-07-16

**Authors:** Suk Yee Lau, Wilson T. Lao

**Affiliations:** 1 Department of Radiology, Wan Fang Hospital, Taipei Medical University, Taipei 116, Taiwan; 2 Department of Radiology, School of Medicine, College of Medicine, Taipei Medical University, Taipei 110, Taiwan

**Keywords:** intrahepatic splenosis, hepatocellular carcinoma, hepatic tumor, splenectomy, splenic trauma

## Abstract

A young adult patient presented to the gastrointestinal outpatient department with a suspected hepatic tumor. The patient was in a traffic accident ten years ago and underwent splenectomy and distal pancreatectomy at another medical institution. The physical examination was unremarkable. The liver function tests and tumor markers were within normal limits, with the alpha-fetoprotein level at 1.38 ng/mL. Both hepatitis B surface antigen and anti-HCV were negative. Based on the clinical history, intrahepatic splenosis was suspected first. Dynamic computed tomography revealed a 2.3 cm lesion exhibiting suspicious early wash-in and early wash-out enhancement patterns. As previous studies have reported, this finding makes hepatocellular carcinoma and metastatic lesions the major differential diagnoses. For further evaluation, dynamic magnetic resonance imaging was performed, and similar enhancing features were observed, along with restricted diffusion. As hepatocellular carcinoma still could not be confidently ruled out, the patient underwent an ultrasound-guided biopsy. The diagnosis of intrahepatic splenosis was confirmed by the pathologic examination. Intrahepatic splenosis is a rare condition defined as an acquired autoimplantation of splenic tissue within the hepatic parenchyma. Diagnosis can be challenging due to its ability to mimic liver tumors in imaging studies. Therefore, in patients with a history of splenic trauma and/or splenectomy, a high index of suspicion and awareness is crucial for accurate diagnosis and for prevention of unnecessary surgeries or interventions.

**Figure 1:**
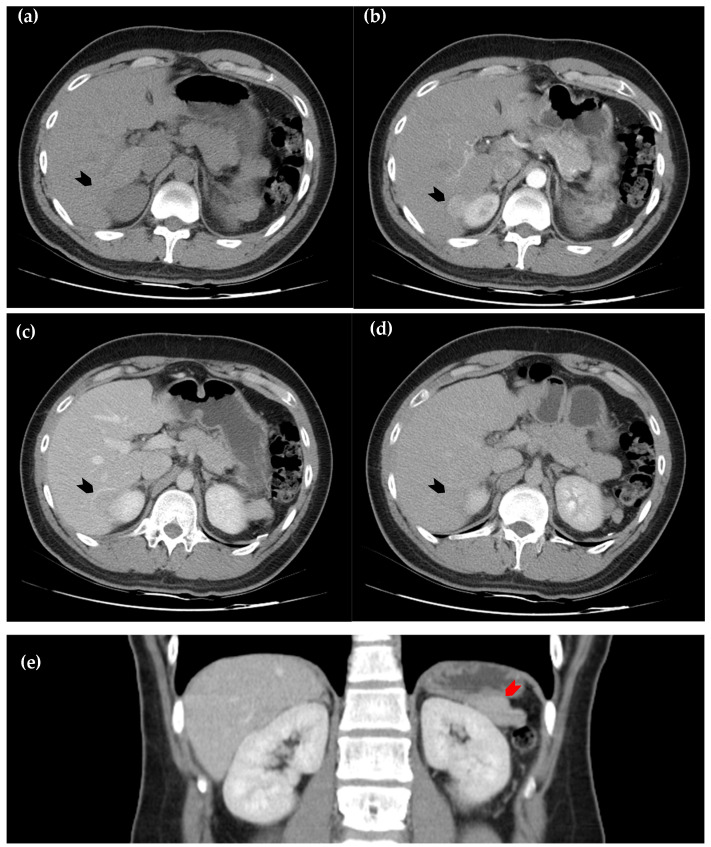
Dynamic computed tomography of the liver. (**a**) Axial view of the pre-contrast sequence shows a slightly hypodense lesion, as compared with the surrounding normal hepatic parenchyma, located in segment six of the liver (black arrowhead); (**b**) axial view of the arterial phase. The lesion exhibits homogeneous enhancement (black arrowhead); (**c**) axial view of the portal phase, in which the lesion becomes relatively hypoattenuating (black arrowhead); (**d**) the axial view of the delayed phase, in which hypoattenuation of the lesion persists but is less prominent than in the portal phase (black arrowhead); (**e**) coronal view of the portal phase, in which a soft tissue lesion at left perinephric area (red arrowhead) demonstrates similar attenuation to the intrahepatic lesion, raising suspicion for splenosis. However, due to the early wash-in and early wash-out enhancement pattern, hepatic tumors such as hepatocellular carcinoma and metastatic lesions remain important differential diagnoses [1,2,3,4].

**Figure 2:**
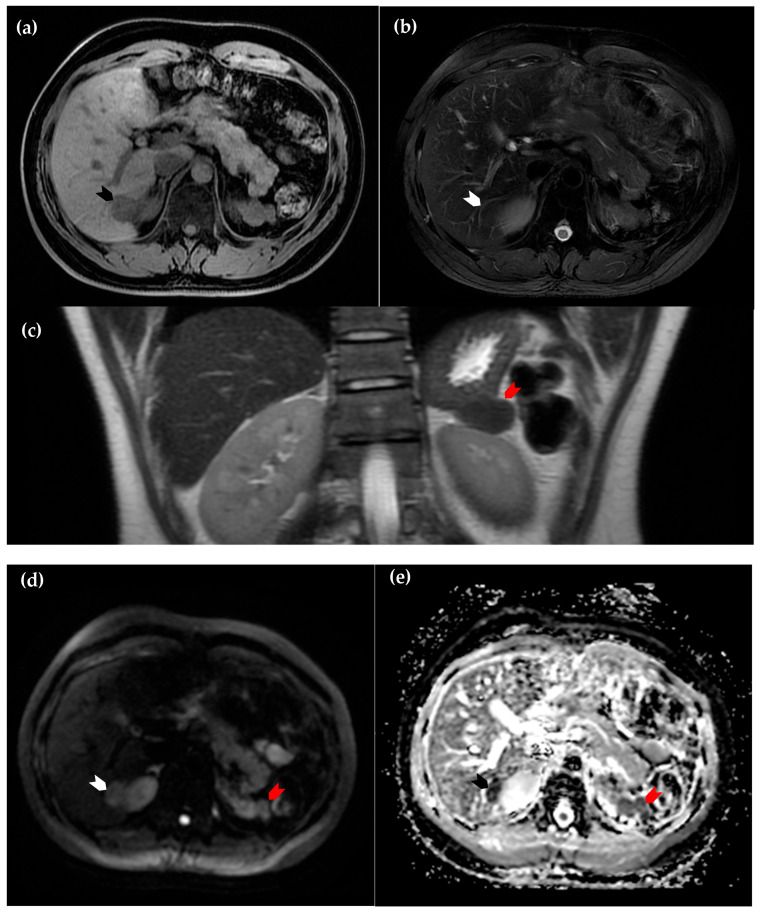
Magnetic resonance imaging of the liver. (**a**) Axial view of the T1 sequence, in which the intrahepatic lesion is hypointense in comparison to surrounding normal hepatic parenchyma (black arrowhead); (**b**) axial view of the T2 sequence, in which the lesion is nearly isointense (white arrowhead); (**c**) coronal view of the T2 sequence. The suspected perinephric splenosis (red arrowhead) again exhibits similar attenuation to the intrahepatic lesion (white arrowhead in (**d**) and black arrowhead in (**e**)), as well as characteristics of restricted diffusion on diffusion-weighted imaging (**d**) and the corresponding apparent diffusion coefficient map (**e**). These findings support the diagnosis of intrahepatic splenosis [2].

**Figure 3:**
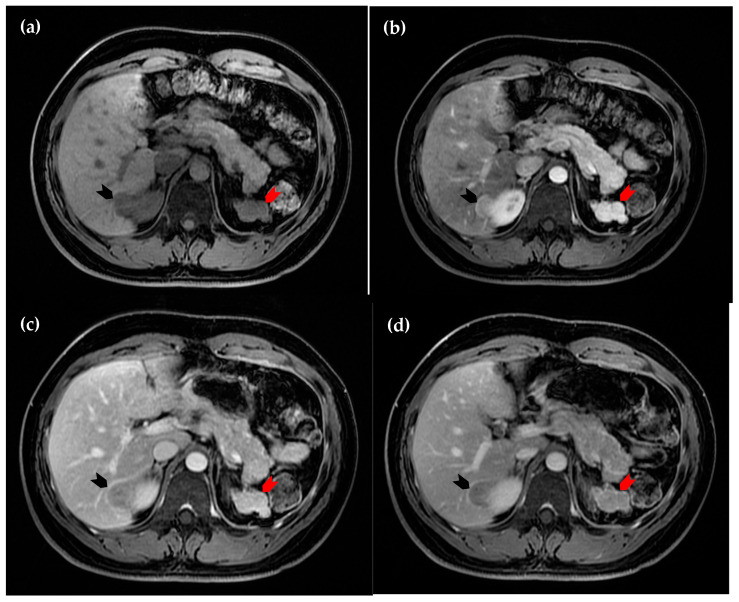
Dynamic magnetic resonance imaging of the liver. (**a**) Axial view of the pre-contrast sequence, in which the intrahepatic lesion is hypointense (black arrowhead); (**b**) axial view of the arterial phase, in which the lesion demonstrates homogeneous hyperintensity (black arrowhead); (**c**) axial view of the portal phase, in which early wash-out of enhancement is observed (black arrowhead); (**d**) axial view of the delayed phase, in which the lesion (black arrowhead) remains more hypointense compared to the surrounding hepatic parenchyma. The perinephric lesion again demonstrates similar attenuation across all sequences (red arrowhead in (**a**–**d**)). However, due to the suspicious enhancement pattern of the intrahepatic lesion, a hepatic tumor cannot be excluded [1,2,3,4].

**Figure 4:**
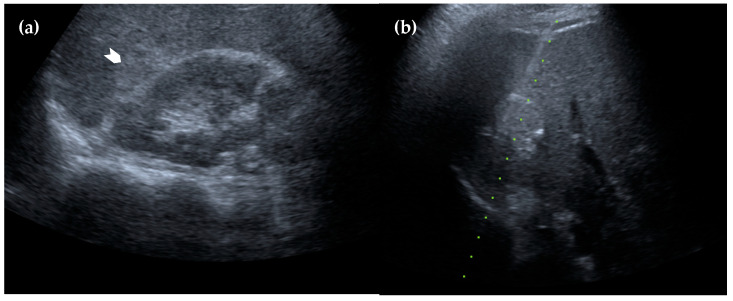
Ultrasonography of the liver. (**a**) The lesion appears homogeneously hyperechoic with a well-defined margin (white arrowhead); (**b**) an ultrasound-guided biopsy was performed, and the pathology results confirmed the diagnosis of intrahepatic splenosis.

## Data Availability

All data are available within the article.
